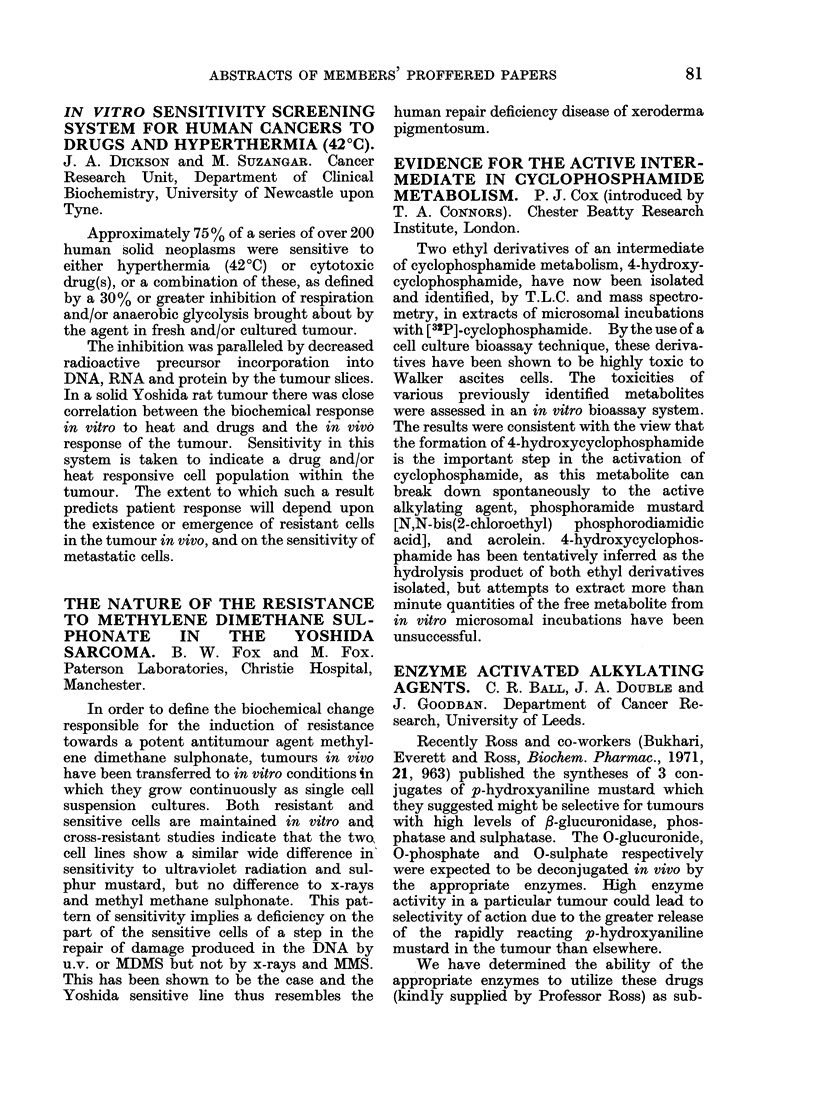# Evidence for the active intermediate in cyclophosphamide metabolism.

**DOI:** 10.1038/bjc.1973.91

**Published:** 1973-07

**Authors:** P. J. Cox


### EVIDENCE FOR THE ACTIVE INTERMEDIATE IN CYCLOPHOSPHAMIDE METABOLISM.

P. J. Cox (introduced by T. A. CONNORS). Chester Beatty Research Institute, London.

Two ethyl derivatives of an intermediate of cyclophosphamide metabolism, 4-hydroxycyclophosphamide, have now been isolated and identified, by T.L.C. and mass spectrometry, in extracts of microsomal incubations with [$^{32}$P]-cyclophosphamide. By the use of a cell culture bioassay technique, these derivatives have been shown to be highly toxic to Walker ascites cells. The toxicities of various previously identified metabolites were assessed in an *in vitro* bioassay system. The results were consistent with the view that the formation of 4-hydroxycyclophosphamide is the important step in the activation of cyclophosphamide, as this metabolite can break down spontaneously to the active alkylating agent, phosphoramide mustard [N,N-bis(2-chloroethyl) phosphorodiamidic acid], and acrolein. 4-hydroxycyclophosphamide has been tentatively inferred as the hydrolysis product of both ethyl derivatives isolated, but attempts to extract more than minute quantities of the free metabolite from *in vitro* microsomal incubations have been unsuccessful.